# Polymorphisms and genetic effects of *PRLR*, *MOGAT1, MINPP1* and *CHUK* genes on milk fatty acid traits in Chinese Holstein

**DOI:** 10.1186/s12863-019-0769-1

**Published:** 2019-08-16

**Authors:** Lijun Shi, Lin Liu, Xiaoqing Lv, Zhu Ma, Yuze Yang, Yanhua Li, Feng Zhao, Dongxiao Sun, Bo Han

**Affiliations:** 1 Department of Animal Genetics, Breeding and Reproduction, College of Animal Science and Technology, Key Laboratory of Animal Genetics, Breeding and Reproduction of Ministry of Agriculture and Rural Affairs, National Engineering Laboratory for Animal Breeding, China Agricultural University, No. 2 Yuanmingyuan West Road, Haidian District, Beijing, 100193 China; 2 Beijing Dairy Cattle Center, Beijing, 100192 China; 3 Beijing General Station of Animal Husbandry, Beijing, 100101 China

**Keywords:** *PRLR*, *MOGAT1*, *MINPP1*, *CHUK*, Genetic effects, Milk fatty acid traits, Chinese Holstein

## Abstract

**Background:**

Our initial genome-wide association study (GWAS) identified 20 promising candidate genes for milk fatty acid (FA) traits in a Chinese Holstein population, including *PRLR*, *MOGAT1, MINPP1* and *CHUK* genes. In this study, we performed whether they had significant genetic effects on milk FA traits in Chinese Holstein.

**Results:**

We re-sequenced the entire exons and 3000 bp of the 5′ and 3′ flanking regions, and identified 11 single nucleotide polymorphisms (SNPs), containing four in *PRLR*, two in *MOGAT1*, two in *MINPP1*, and three in *CHUK*. The SNP-based association analyses showed that all the 11 SNPs were significantly associated with at least one milk FA trait (*P* = 0.0456 ~ < 0.0001), and none of them had association with C11:0, C13:0, C15:0 and C16:0 (*P* > 0.05). By the linkage disequilibrium (LD) analyses, we found two, one, one, and one haplotype blocks in *PRLR*, *MOGAT1*, *MINPP1*, and *CHUK*, respectively, and each haplotype block was significantly associated with at least one milk FA trait (*P* = 0.0456 ~ < 0.0001). Further, g.38949011G > A in *PRLR*, and g.111599360A > G and g.111601747 T > A in *MOGAT1* were predicted to alter the transcription factor binding sites (TFBSs). A missense mutation, g.39115344G > A, could change the PRLR protein structure. The g.20966385C > G of *CHUK* varied the binding sequences for microRNAs. Therefore, we deduced the five SNPs as the potential functional mutations.

**Conclusion:**

In summary, we first detected the genetic effects of *PRLR*, *MOGAT1, MINPP1* and *CHUK* genes on milk FA traits, and researched the potential functional mutations. These data provided the basis for further investigation on function validation of the four genes in Chinese Holstein.

**Electronic supplementary material:**

The online version of this article (10.1186/s12863-019-0769-1) contains supplementary material, which is available to authorized users.

## Background

For dairy breeding, the weight of milk components has attracted more and more attention. Fatty acids (FAs) are the important components of milk fat, and the milk fat contains 70% of saturated fatty acids (SFAs) and 30% unsaturated fatty acids (UFAs) [1]. While, the ideal nutritional milk fat contain 10% polyunsaturated fatty acids, 82% monounsaturated fatty acids and 8% SFAs, that can not be accomplished by modifying diets of lactating cows [2]. Therefore, researches paid more attentions on the genetic improvement for milk FA traits in dairy cattle, and studies showed that the heritability of SFA and UFA were 0.14–0.33 and 0.08–0.29, respectively, in Holstein cows [3–7].

There is a growing number of genome-wide association studies (GWASs) on milk FA traits, and many candidate genes were identified [8–12]. Our previous GWAS [12] showed that four significant single nucleotide polymorphisms (SNPs), BTB-01423653, BTB-01423676, Hampmap30570-BTA-152778 and ARS-BFGL-NGS-17676, with the distance of 55 ~ 495 kb from *PRLR* (Prolactin receptor) were associated with C18index ($\frac{\mathbf{C18}:\mathbf{1}}{\mathbf{C18}:\mathbf{1}+\mathbf{C}\mathbf{1}\mathbf{8}:\mathbf{0}}\times \mathbf{1}\mathbf{0}\mathbf{0}$), SFA, UFA and SFA/UFA (*P* = 2.45E-05 ~ 2.55E-06); ARS-BFGL-NGS-13938, 19.3 kb far away from *MOGAT1* (Monoacylglycerol O-acyltransferase 1), was significantly associated with UFA (*P* = 2.25E-05); BTA-111275-no-rs within the *MINPP1* (Multiple inositol-polyphosphate phosphatase 1) was significantly associated with C12:0 (*P* = 2.39E-05); and Hapmap46411-BTA-15820 within the *CHUK* (Conserved helix-loop-helix ubiquitous kinase) had strong associations with C14:1 (*P* = 8.29E-06) and C14index ($\frac{\mathbf{C14}:\mathbf{1}}{\mathbf{C14}:\mathbf{1}+\mathbf{C}\mathbf{1}\mathbf{4}:\mathbf{0}}\times \mathbf{1}\mathbf{0}\mathbf{0}$ ; *P* = 1.10E-08). Thus, the four genes were considered as the promising candidates for milk FA traits in Chinese Holstein.

*PRLR* during the pregnancy may be associated with mammary development [13], and it can guide and maintain mammary epithelial cells for continuous lactation during milking of dairy cows [14]. *MOGAT1*catalyzes the conversion of monoacylglycerols to diacylglycerols, the precursor of several physiologically important lipids such as phosphatidylethanolamine and triacylglycerol [15]. *MOGAT1* expression is inversely correlated with lipolytic rates, and its suppression increases basal lipolytic activity [16]. *MINPP1* is a stress protein and it can interact with the glucose-1-phosphate to induce apoptosis [17, 18] and human *MINPP1* plays a role in differentiation and apoptosis [19]. *CHUK* gene is also named IKKA gene, and IKKα is normally an activator of the transcription factor nuclear factor-κB, and it leads to potent activation of SREBP-mediated lipogenesis in the context of hepatitis C virus infection [20].

In this study, we further explored the polymorphisms and genetic effects of *PRLR*, *MOGAT1*, *MINPP1* and *CHUK* genes on milk FA traits, and searched the potential functional mutations.

## Results

### SNPs identification

In this study, we identified four, two, two, and three SNPs in *PRLR*, *MOGAT1*, *MINPP1*, and *CHUK* genes, respectively (Additional file 2: Table S2). For *PRLR*, two SNPs (g.38948871C > T and g.38949011G > A) were in the 5′ flanking region, and two SNPs (g.39115344G > A and g.39115345 T > C) were in the exon 4. In *MOGAT1*, both g.111599360A > G and g.111601747 T > A were in the 5′ flanking region. The g.9206582C > T and g.9207070A > G were observed in the 3′ UTR and the intron 5 of *MINPP1*, respectively. For *CHUK*, g.21008688G > T was in the 5′ flanking region, and two SNPs (g.20966385C > G and g.20966354C > T) were in the 3′ UTR. Out of these SNPs, g.39115344G > A in *PRLR* was a missense mutation with the substitution of amino acid from serine to asparagine when the allele mutated from G to A.

### Associations between SNPs/haplotype blocks and milk fatty acids

We performed the association analyses between the total 11 SNPs and 23 milk FA traits, and the results were shown in Additional file 3: Table S3. For *PRLR* gene, g.38948871C > T was significantly associated with C6:0 (*P* = 0.0027) and UFA (*P* = 0.0364); g.38949011G > A was significantly associated with C8:0 (*P* = 0.0108); g.39115344G > A was significantly associated with C6:0, C8:0, C14:0, C17:1, C17index, SFA, and UFA (*P* = 0.022 ~ < 0.0001); and g.39115345 T > C was significantly associated with C6:0, C8:0, C10:0, C14:0, C18index, C17index, SFA, and UFA (*P* = 0.0456 ~ < 0.0001). For *MOGAT1* gene, the two SNPs (g.111599360A > G and g.111601747 T > A) were significantly associated with C8:0 (*P* = 0.0001 and *P* < 0.0001), and g.111599360A > G was also significantly associated with C18:0 (*P* = 0.0058) and C17index (*P* = 0.0153). Both g.9206582C > T and g.9207070A > G of *MINPP*1 gene had significant associations with C6:0, C8:0, C10:0 and C17:0 (*P* = 0.0271 ~ < 0.0001), and g.9206582C > T was also significantly associated with C20:0 (*P* = 0.0436). The association analyses results of three SNPs (g.21008688G > T, g.20966385C > G and g.20966354C > T) of the *CHUK* showed that they were all significantly associated with C8:0, C10:0, C12:0, C14:0, C16:1, C17:0, C17:1, C18:0, C16index, and C17index (*P* = 0.0423 ~ < 0.0001). In addition, g.21008688G > T was strongly associated with C18index, C20:0, and UFA (*P* = 0.0099 ~ 0.0014); g.20966385C > G was strongly associated with C14:1 (*P* = 0.0368) and C14index (*P* < 0.0001); and g.20966354C > T had significant associations with C6:0, C18:1cis-9, C18index, C20:0, SFA, UFA, and SFA/UFA (*P* = 0.0114 ~ < 0.0001). Correspondingly, the significances of additive (а), dominant (d) and substitution (α) effects for the 11 SNPs with milk FA traits were shown in Additional file 4: Table S4.

**Fig. 1:**
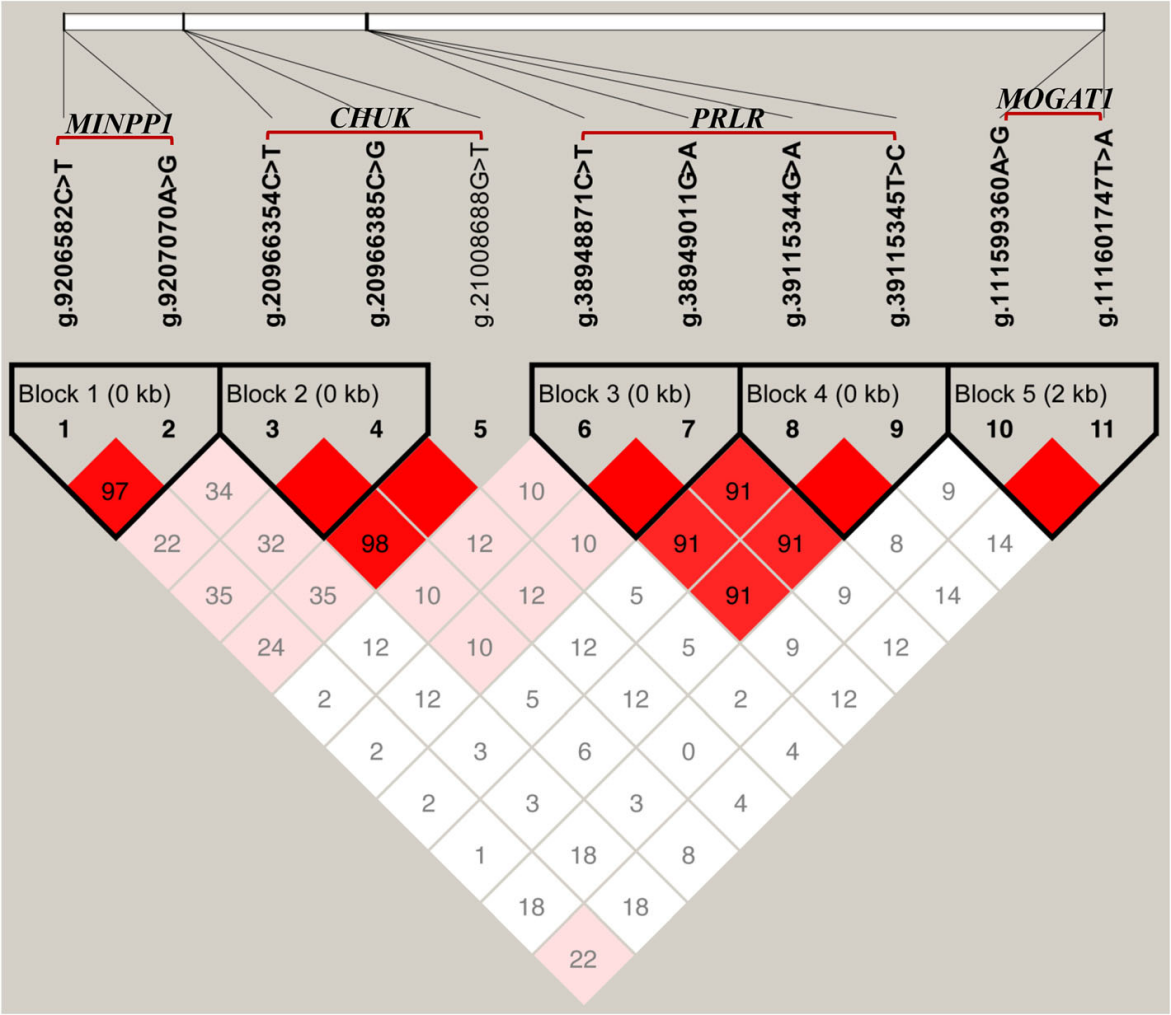
The linkage disequilibrium (LD; D′ =0.97 ~ 1.00) estimated among the SNPs in *PRLR*, *MOGAT1*, *MINPP1* and C*HUK* genes. Haplotype block 1 included g.38948871C > T and g.38949011G > A in *PRLR*; haplotype block 2 included g.39115344G > A and g.39115345 T > C in *PRLR*; haplotype block 3 included g.111599360A > G and g.111601747 T > A in *MOGAT1*; haplotype block 4 included g.9206582C > T and g.9207070A > G in *MINPP1*; and haplotype block 5 included g.20966385C > G and g.20966354C > T in C*HUK*. D′ is the value of D prime between the two loci

We found five haplotype blocks (D′ = 0.97 ~ 1.00; Fig. 1) in the study, including two in *PRLR*, one in *MOGAT1*, one in *MINPP1*, and one in *CHUK*. By haplotype-based association analyses (Additional file 5: Table S5), we found that the block 1 in *PRLR* was significantly associated with C6:0 (*P* = 0.0389); block 2 in *PRLR* was significantly associated with C6:0, C14:0, C17:1, C17index, SFA and UFA (*P* = 0.0392 ~ 0.0001); block 3 in *MOGAT1* had significant associations with C8:0, C16:1, C18:0 and C17index (*P* = 0.0364 ~ < 0.0001); block 4 in *MINPP1* had strong associations with C6:0, C8:0, C10:0, C18:1cis-9, C20:0, C17index, and UFA (*P* = 0.0306 ~ < 0.0001); and block 5 in *CHUK* was strongly associated with C6:0, C8:0, C10:0, C14:0, C16:1, C17:0, C18:0, C18:1cis-9, C18index, C20:0, C14index, C16index, C17index, SFA, UFA, and SFA/UFA (*P* = 0.0256 ~ < 0.0001).

### Functional variation caused by SNPs of *PRLR*, *MOGAT1*, *MINPP1* and *CHUK*

**Fig. 2:**
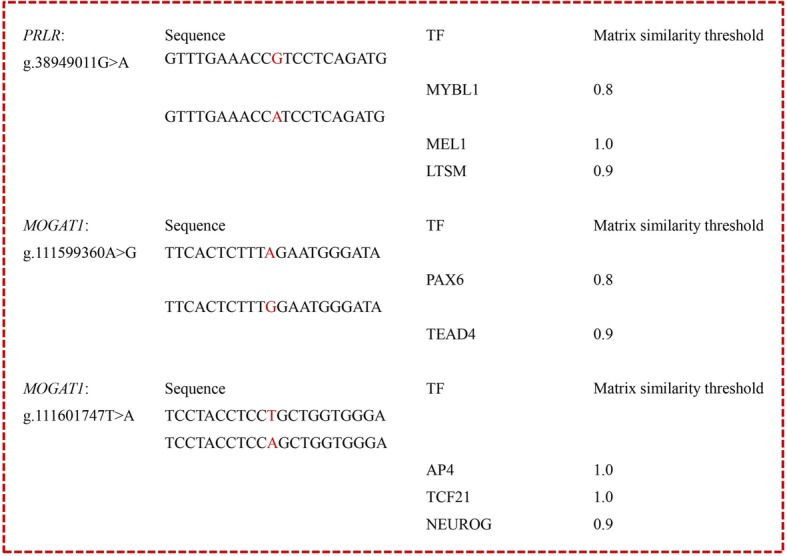
Changes of transcripton factor binding sites caused by the SNPs in 5′ flanking regions. The SNPs in sequences were highlighted in red

We used the Genomatix software to predicted the changes of TFBSs for all the five SNPs in 5′ flanking region of the four genes, and found that totally three SNPs (g.38949011G > A, g.111599360A > G and g.111601747 T > A) could alter the TFBSs (Fig. 2). The allele G of g.38949011G > A created a BS for the transcription factor MYBL1 (V-myb avian myeloblastosis viral oncogene homolog-like 1; MST = 0.8), and the allele A created the BSs for MEL1 (MDS1/EVI1-like gene 1; MST = 1.0) and LTSM (LTSM elements with 8 bp spacer; MST = 0.8). Alleles A and G of g.111599360A > G created the BSs for PAX6 (Pax-6 paired domain binding site; MST = 0.8) and TEAD4 (TEA domain family member 4; MST = 0.9), respectively. The allele A of g.111601747 T > A created three TFBSs, namely, AP4 (Activating enhancer binding protein 4; MST = 1.0), TCF21 (Transcription factor 21; MST = 1.0) and NEUROG (Neurogenin 1 and 3 binding sites; MST = 0.9).

We further used the SOPMA to predict the protein secondary structure variation for the missense mutation (g.39115344G > A) in *PRLR*, and found that the PRLR protein secondary structure altered from serine (AGT) to asparagine (AAT). It showed that the α-helix was from 16.70 to 14.97%, the extended strand from 21.86 to 21.69%, the β-turn from 4.65 to 3.79%, and the random coil from 56.80 to 59.55%.

In addition, we used the position information of all the SNPs in the 3′ UTR to confirm that whether the SNPs altered the BSs for the microRNAs according to the TargestScanHuman database, and found that the allele C of g.20966385C > G in the 3′ UTR of *CHUK* was located in the seed region for targeting the specific microRNAs, bta-miR-2392, bta-miR-2434/3602 and bta-miR-2395.

## Discussion

The *PRLR*, *MOGAT1, MINPP1* and *CHUK* genes were considered as the promising candidates for milk FA traits in our previous GWAS [12], and their polymorphisms and genetic effects were determinated in this study.

In recent years, the association between the gene polymorphism and milk production traits in dairy cattle has become a hotspot [21–23]. In this study, we found that each SNP of the *PRLR*, *MOGAT1*, *MINPP1*, and *CHUK* genes had significant association with at least one milk FA trait. Haplotypes formed by the SNPs have important implications for identifying associations with complex traits [24]. For the identified SNPs, we estimated their LD, and found that they were highly linked, which might be due to the properties of each SNP, such as the frequency and population history [25]. The haplotype-based association analyses showed that each haplotype block was also significantly associated with at least one milk FA trait.

From the KEGG database (https://www.genome.jp/kegg/pathway.html), we found that *PRLR* gene is involved in PI3K-AKT (ko04151) and Jak-STAT (ko04630) signaling pathways, which were identified to be associated with lipid metabolism [26, 27]. In this study, g.38949011G > A in the 5′ flanking region of *PRLR* altered the TFBSs, in which, the allele G created a BS for and the MYBL1, and the allele A created the BSs for MEL1 and LTSM. As we know, transcription factors (TFs) are the sequence-specific DNA-binding proteins that can regulate gene transcription, and genomic locations at which TFs interact with DNA are considered as TFBSs [28]. Some studies showed that TFs can play the important roles in gene expression [29, 30]. MYBL1 as a DNA-binding TF can bind the *mim-1* promoter and to activate its expression to regulate the oncogenesis [31], and MEL1 can stabilize the inactive Smad3-SKI complex on the promoter of TGF-β target genes to inhibit TGF-β signaling [32]. LTSM can enhance the transcriptional activity of the promoters in dependency of the distance from the transcription start site [33]. Hence, we deduced that these TFs might regulate *PRLR* gene expression to impact the components of milk FAs. In addition, we found a missense mutation in *PRLR* gene, g.39115344G > A, altered the protein secondary structure. Proteins are the utmost multi-purpose macromolecules (i.e., main catalysts, structural elements, signaling messengers and molecular machines of biological tissues) [34], which play a crucial function in many aspects of biological processes [35]. The protein structure plays a decisive role in function of protein, for example, a study reports that the β-turn is essential for the structure and function of proteins [36]. The prediction of protein structure from amino acid sequences is one of the most vital problems in molecular biology, and the fundamental elements of the protein secondary structure are α-helix, extended strand, β-turn, and coils [37]. In this study, α-helix, extended strand, β-turn, and random coil were all changed because of the mutation from G to A in g.39115344G > A, indicating that this missense mutation might affect the function of PRLR through changing the protein secondary structure.

*MOGAT1* gene is involved in the glycerolipid metabolism (ko00561), and codes the MGAT (monoacylglycerol acyltransferases) enzyme, which is active in human liver and its activity can represent a viable target for pharmacologic intervention to treat nonalcoholic fatty liver disease [38]. A study reported that up-regulation of MOGAT1 gene can mediate hepatic steatosis by increasing intracellular diacylglycerol content [39]. In this study, g.111599360A > G in 5′ flanking region of *MOGAT1* was predicted to create the TFBSs for PAX6 with the allele A and TEAD4 with the allele G. PAX6 can respectively down-regulate Sox3 and up-regulate Lhx9 in the Pax6-mutant cortex to exert its effects at the molecular level during murine forebrain neurogenesis [40]. TEAD4 can directly induce *Myogenin*, *CDKN1A* and *Cavelin3* expression to promote myoblast differentiation [41]. Mutation of TAED4 at either site can decrease its occupancy on the promoter region of target genes, and largely impair the target gene transcription to inhibit the growth and colony formation of gastric cancer cell HGC-27 [42]. By association analyses, the cows with AA genotype of g.111599360A > G had higher C8:0 than that with GG, implicating that PAX6 might enhance the content C8:0 by regulating the target gene *MOGAT1*. While, the TEAD4 might regulate *MOGAT1* to reduce C8:0 by binding the G allele. In addition, g.111601747 T > A in the 5′ flanking region of *MOGAT1*, created the BSs for three TFs, namely, AP4, TCF21, and NEUROG. It is reported that AP4 can up-regulate the expression of *LAPTM4B* to promote cell growth, migration, invasion, and cisplatin resistance in breast cancer [43]. AP4-geminin complex suppresses the precocious expression of target genes in fetal brain [44]. TCF21 plays a repression role for its target gene to affect the phenotypes [45, 46]. Over-expression of NEUROG can override the pluripotency-specific gene network and force human embryonic stem cells to differentiate into neurons [47]. The cows with AA genotype of g.111601747 T > A had significantly lower C8:0, suggesting that the three TFs (AP4, TCF21 and NEUROG) might work together to regulate the expression of *MOGAT1* gene to finally decrease the C8:0.

*MINPP1* is involved in the glycolysis/gluconeogenesis (ko00010) and inositol phosphate metabolism (ko00562) signaling pathways. Glycolysis can completely bypass 3-phosphoglycerate through that *MINPP1* converts 2,3-bisphosphoglycerate to 2-phosphoglycerate, which activates the AMPK cascade [48]. Furthermore, AMPK can stimulate the fatty acid oxidation [49]. In this study, we identified g.9207070A > G in the intron 5 of the *MINPP1* gene. In 1987, the first finding that introns can increase the gene expression was found in maize [50]. Introns include the regulatory regions, that can conferred developmental and cell-specific expression of a gene reside [51]. The rs734553 located on the intron 7 of *SLC2A9* in human is greatly related with serum uric acid of healthy individuals with normal renal function, thus it is powerful for prediction of chronic kidney disease progression [52]. Our association analyses showed that the cows with GG genotype of g.9207070A > G had significantly higher C6:0, and lower C17:0. We herein concluded that the intron mutation might be able to affect the milk FA traits that deserved the further validation.

*CHUK* gene is involved in the MAPK (ko04010), mTOR (ko04150), PI3K-Akt (ko04151), and Adipocytokine (ko04920) signaling pathways associated with lipid metabolism. In 2012, the associations between a SNP (rs11597086) of the human *CHUK* and lipid phenotype were identified [53]. In this study, the allele C of g.20966385C > G in the 3′ UTR of *CHUK* is in the seed sequences for targeting the microRNAs (bta-miR-2392, bta-miR-2434/3602 and bta-miR-2395). MicroRNA is a class of gene expression regulating factors and plays an important role in maintaining genome stability, regulating growth and development, and other biological processes [54, 55]. Some studies reported that microRNA regulates the fat metabolism and lipid metabolism disorders through the targeting genes, for example, miR-196 may be related to the gene expression of the homologous genes in subcutaneous adipose tissue and lipid distribution [56]. For the three microRNAs, bta-miR-2392, bta-miR-2434/3602 and bta-miR-2395, there have not been studied to reveal the regulatory function in cattle until now. In human, the miR-2392 can regulate its target gene *MAML3* and *WHSC1* to suppress metastasis and epithelial-mesenchymal transition in gastric cancer [57]. Our association analyses showed that the cows with CC genotype of g.20966385C > G had significantly higher C14:1, C17:0, C18:0 and C14inex, and lower C14:0, C16:1, C16index and C17index, indicating that the three microRNAs might regulate the expression of *CHUK* to affect the milk FA traits.

In dairy cattle breeding, the integration of DNA marker technology and genomics into the traditional evaluation system decreased generation interval and increased the selection accuracy, so that the cost of progeny testing was reduced [58]. Here, we found significant SNPs for milk FAs in dairy cattle, which could be used as the genetic markers for the genomic selection to improve the accuracy of selection and lower the breeding cost. This study provided the evidence for associations of *PRLR*, *MOGAT1*, *MINPP1* and *CHUK* genes with milk FAs, and the in-depth study should be performed to verify the regulatory mechanism of these genes for milk FAs in dairy cattle by biotechnologies, such as RNA interference and gene editing.

## Conclusions

Our findings first confirmed the genetic effects of *PRLR*, *MOGAT1*, *MINPP1* and *CHUK* genes on milk FAs in Chinese Holstein. Three SNPs, g.38949011G > A of *PRLR*, g.111599360A > G and g.111601747 T > A of *MOGAT1*, might be the functional mutations by changing the promoter activities. In addition, the missense mutation in *PRLR*, g.39115344G > A, changed the protein secondary structure was suggested to be a critical mutation to affect the PRLR protein function. Furthermore, g.20966385C > G in 3′ UTR of *CHUK* varied the binding sequences for microRNAs that could regulate the gene expression of *CHUK*. This study provided the basis for further investigation on function validation of *PRLR*, *MOGAT1*, *MINPP1* and *CHUK* genes, and the potential functional mutations might serve as genetic markers to apply to the breeding program for milk FA traits in Chinese Holstein.

## Methods

### Animals and phenotypic data

A total of 1065 Chinese Holstein cows belonging to 44 sire families were collected from 23 dairy farms of Beijing Sanyuanlvhe Dairy Farming Center (Beijing, China), where the standard performance testing for dairy herd improvement (DHI) has been regularly conducted since 1999. These cows were full blood of Chinese Holstein breed that were originated from cross-breeding between the Chinese Yellow cattle and European Holstein, over the past 100 years, by continuous import of foreign Holstein bulls, semen and embryos, mainly from USA and a few from Canada and Europe, which were directly used to AI or used to cross Chinese Holstein cows through planned mating to generate breeding bulls [59]. All the cows were fed with the same regular total mixed ration composed of concentrated feed and coarse fodder. We obtained 50 ml milk samples during November to December of 2014 to measure the DHI, and then the remaining milk were frozen in the − 20 °C to be used for detecting the milk FAs in the laboratory of the Beijing Dairy Cattle Center (www.bdcc.com.cn). We used gas chromatography to measure the 16 FAs (C6:0, C8:0, C10:0, C11:0, C12:0, C13:0, C14:0, C15:0, C16:0, C17:0, C18:0, C20:0, C14:1, C16:1, C17:1, and C18:1cis-9) in the milk samples according to the descriptions of procedures in the previous studies [12, 60], and calculated C14index, C16index, C17index and C18index using the formula [61]: $\frac{\mathrm{cis}-9\ \mathrm{unsaturated}}{\mathrm{cis}-9\ \mathrm{unsaturated}+\mathrm{saturated}}\times 100$. In addition, we summarized SFA, UFA and SFA/UFA.

### DNA extraction and SNP identification

We used the TIANamp Blood DNA kit (Tiangen, Beijing, China) and salt-out procedure to extract the genomic DNA form the blood samples of 1065 Chinese Holsteins and the semen samples of 44 sires, respectively. Then the quantity and quality of the genomic DNAs were measured by NanoDrop 2000 Spectrophotometer (Thermo Scientific, DE, USA) and gel electrophoresis, respectively.

We designed a total of 85 primers (Additional file 1: Table S1) in the entire exons with their partial adjacent introns, and 3000 bp of 5′ and 3′ flanking regions of the *PRLR*, *MOGAT1*, *MINPP1*, and *CHUK* genes using the Primer 3 (http://bioinfo.ut.ee/primer3-0.4.0/) according to their bovine genomic sequences (GenBank accession no. AC_000177.1, AC_000159.1, AC_000183.1 and AC_000183.1). The primers were synthesized in Beijing Genomics Institute (BGI, Beijing, China). The 44 semen DNAs were randomly divided into two DNA pools (22 DNA in each pool) for the PCR amplification, and the concentration of each DNA was 50 ng/μL. PCR conditions were as follows: initial denaturation at 94 °C for 5 min, followed by 35 cycles at 94 °C for 30s; 60 °C for 30s; 72 °C for 30s; and a final extension at 72 °C for 7 min. Afterwards, we sequenced the PCR products using ABI3730XL DNA analyzer (Applied Biosystems, Foster, CA, USA), and aligned the sequences with the bovine reference sequence (UMD 3.1) by the BLAST (http://blast.ncbi.nlm.nih.gov/Blast.cgi) for identifying the potential SNPs.

### Genotyping and linkage disequilibrium (LD) analyses

Genotypes of the identified SNPs were obtained from 1065 cows with Sequenom MassArray by matrix-assisted laser desorption/ionization time of flight mass spectrometry (MALDI-TOF MS, Sequenom MassARAY, Bioyong Technologies Inc., HK). In addition, we used the Haploview 4.1 (Broad Insititute, Cambridge, MA, USA) to analyze the LD extent among the identified SNPs.

### Association analyses

The association analyses between each SNP/haplotype block and 23 milk FA traits were conducted with SAS9.2 (SAS Institute Inc., Cary, NC, USA) using the following model:

$$ {Y}_{ijklm}=\upmu +{G}_i+{h}_j+{l}_k+{a}_l+\mathrm{b}\times {M}_m+{e}_{ijklm} $$

For each trait, *Y*_*ijklm*_ was the phenotypic value of the 1065 cows; μ was the overall mean; *G*_*i*_, *h*_*j*_ *l*_*k*_ and *M*_*m*_ were the fixed effects of the genotypes or haplotypes, farm (23), stage of lactation (4) and calving months (293), respectively; *a*_*l*_ was the random polygenic effect; b represented the regression coefficient of covariate M; and *e*_*ijklm*_ was the random residual. We considered a significant association when the *P* was less than 0.05/N, where N was the number of genotypes or haplotype combinations. In addition, we calculated the additive effect (a), dominant effect (d), and substitution effect (α) according to the formulas by Falconer & Mackay [62]: *a* = (*AA* − *BB*)/2, *d* = *AB* − (*AA* + *BB*)/2 and *a* = *a* + *d*(*q* − *p*), where AA, AB and BB represent the square means of milk FA traits corresponding to the genotypes, *p* and *q* refer to the allele frequencies of corresponding loci.

### Biological function prediction

We used the Genomatix software (http://www.genomatix.de/cgi-bin/welcome/welcome.pl?s=d1b5c9a9015b02bb3b1a806f9c03293f) to predict whether the SNPs in the 5′ flanking region of the *PRLR*, *MOGAT1*, *MINPP1* and *CHUK* genes altered the transcription factor binding sites (TFBSs; matrix similarity threshold, MST > 0.8). Furthermore, we explored the changes of protein secondary structure for missense mutation by the SOPMA software (https://prabi.ibcp.fr/htm/site/web/services/secondaryStructurePrediction#SOPMA). For the SNPs in the 3′ untranslated region (UTR), we aligned them to the TargetScanHuman database (http://www.targetscan.org/vert_71/) for researching whether they changed the binding sites (BSs) of seed sequences with the microRNAs.

## Additional files

Additional file 1: **Table S1.** Details of PCR primers of *PRLR*, *MOGAT1*, *MINPP1*, and *CHUK* genes. (XLSX 20 kb)

Additional file 2: **Table S2.** Information about the 11 identified SNPs. (XLSX 10 kb)

Additional file 3: **Table S3.** Associations of 11 SNPs of *PRLR*, *MOGAT1*, *MINPP1* and *CHUK* genes with fatty acid traits in Chinese Holstein (LSM ± SE). (XLSX 26 kb)

Additional file 4: **Table S4.** Additive (a), dominant (d), and allele substitution (α) effects of 11 SNPs on milk fatty acid traits in Chinese Holstein cows. (XLSX 18 kb)

Additional file 5: **Table S5.** Associations of the haplotype blocks with milk fatty acid traits in Chinese Holstein (LSM ± SE). (XLSX 19 kb)

## Data Availability

All relevant data are available within the article and its additional files.

## Abbreviations

a: Additive effect
BS: Binding site
CHUK: Conserved helix-loop-helix ubiquitous kinase
d: Dominant effect
DHI: Dairy herd improvement
FA: Fatty acids
GWAS: Genome-wide association study
LD: Linkage disequilibrium
MINPP1: Multiple inositol-polyphosphate phosphatase 1
MOGAT1: Monoacylglycerol O-acyltransferase 1
MST: Matrix similarity threshold
PRLR: Prolactin receptor
SFA: Saturated fatty acids
SNP: Single nucleotide polymorphism
TF: Transcription factor
TFBS: Transcription factor binding site
UFA: Unsaturated fatty acids
UTR: Untranslated region
α: Substitution effect
